# What is the predictive value of preoperative CA 125 level on the survival rate of type 1 endometrial cancer?

**DOI:** 10.3906/sag-2005-331

**Published:** 2021-02-26

**Authors:** Şafak YILMAZ BARAN, Songül ALEMDAROĞLU, Gülşen DOĞAN DURDAĞ, Seda YÜKSEL ŞİMŞEK, Filiz AKA BOLAT, Fatih KÖSE, Hüsnü ÇELİK

**Affiliations:** 1 Department of Obstetrics and Gynecology, Başkent University, Dr. Turgut Noyan Application and Research Center, Adana Turkey; 2 Department of Pathology, Başkent University, Dr. Turgut Noyan Application and Research Center, Adana Turkey; 3 Department of Medical Oncology, Başkent University, Dr. Turgut Noyan Application and Research Center, Adana Turkey

**Keywords:** Cancer antigen 125 (CA 125), cut-off value, endometrial carcinoma, prognosis, survival

## Abstract

**Background/aim:**

To investigate the utility of preoperative serum cancer antigen 125 (CA 125) levels in type 1 endometrial carcinoma (EC) as a marker for determining poor prognostic factors and survival.

**Material and methods:**

All patients with endometrial cancer, who had been treated between 2012 and 2020, were retrospectively reviewed, and finally, 256 patients with type 1 endometrium carcinoma were included in the study. The relationship between the clinicopathological characteristics, CA 125 level, and survival rates were analyzed. The cut-off value for the preoperative serum CA 125 level was defined as 16 IU/L.

**Results:**

The median serum CA 125 levels were significantly higher in patients with deep myometrial invasion, lymph node metastasis, lymphovascular space invasion, cervical stromal and adnexal involvement, advanced stage, positive peritoneal cytology, recurrence, and adjuvant therapy requirement. Serum CA 125 cut-off values determined according to clinicopathologic factors ranged from 15.3 to 22.9 IU/L (sensitivity 61%–77%, specificity 52%–73%). The disease-specific survival rate was significantly higher in patients with CA 125 levels < 16 IU/L (P = 0.047).

**Conclusion:**

The data showed that choosing a lower threshold value for the CA 125 level (16 IU/L) instead of 35 IU/L, could be more useful in type 1 EC patients with negative prognostic factors.

## 1. Introduction

Endometrial carcinoma (EC) is the most frequent gynecological malignancy in developed countries [1]. Most cases are diagnosed in the early stages, and surgery remains the mainstay of treatment. Based on histopathology and pathological staging, patients are further classified into low-, intermediate-, and high-risk endometrial cancer groups [2]. These classifications help to guide clinicians in the adjuvant chemotherapy and radiotherapy decision. Beyond the stage and tumor histology, novel molecular characterization can also support decision-making, but more data are needed, and these tests are not available for common use. Therefore, it is important to identify patients with a high relapse rate, even before primary surgery, to make a treatment and surveillance plan for these patients [3].

Previous studies have shown that the level of preoperative serum cancer antigen 125 (CA 125), which is known as an epithelial surface tumor antigen, is a useful marker in predicting poor prognostic factors, such as extrauterine spread of disease and recurrence in patients with endometrial cancer [4,5]. The CA 125 test is widely used and accessible for patients in the gynecology unit. Preoperative assessment of CA 125 can be used as an additional tool in preoperative risk stratification to delineate patients with poor outcomes [6,7]. Although there are studies suggesting a cut-off of 35 IU/L as the preoperative CA 125 level in determining prognostic factors and survival in EC [4,8–11], many different cut-off values have been used [12–19]. However, the preoperative serum CA 125 level in endometrial cancer is still unclear and controversial [9]. 

In the current study, the medical records of endometrial cancer patients over the last 8 years were reviewed and retrospectively analyzed to evaluate the utility of preoperative serum CA 125 levels in type 1 EC as a marker for determining poor prognostic factors and survival.

## 2. Materials and methods 

The medical records of 452 EC patients who were treated at our tertiary gynecologic oncology center, between February 2012 and February 2020, were analyzed. Overall, 13 patients who had undergone primary surgery at different centers, while final surgical staging was performed in our clinic. A total of 95 EC patients with type 2 histology, 3 patients who had synchronous endometrium and ovarian cancer, 39 patients whose postoperative follow-up period was less than 3 months, and 46 patients who did not have preoperative serum CA 125 testing were excluded in the study. Finally, 256 patients with documented preoperative serum CA 125 levels in EC, with type 1 histology (endometrioid and mucinous adenocarcinoma), were included in the final analysis. 

All of the patients underwent full surgical staging, as total hysterectomy/bilateral salpingo-oophorectomy, by the same gynecologic oncology team. Systematic lymphadenectomy (pelvic ± para-aortic) was performed on all of the patients, except those with grade 1–2 tumors, with a tumor size <2 cm, and <50% myometrial invasion (MI), according to preoperative endometrial sampling or intraoperative frozen evaluation [20]. Moreover, based on the experience of the surgeon, lymphadenectomy was performed on patients with grade 2 tumors to avoid undertreatment. 

 The staging was determined based on surgical and pathological assessment according to the International Federation of Gynecology and Obstetrics (FIGO) 2009 guidelines [21]. Adjuvant treatment decision was made by a multidisciplinary team on a multidisciplinary tumor board [3]. After definitive treatment, all of the patients were followed-up once every 3 months for 2 years, once every 6 months for 2–5 years, and annual follow-up examinations were performed each year following that. 

For each patient, clinicopathological and demographic characteristics, including preoperative serum CA 125 values, surgical procedures performed, posttreatment findings on physical examination, imaging, and cytological results, were investigated. Based on the histopathologic examination results, the size of the tumor, histologic type, grade, MI depth, peritoneal cytology, lymphovascular space invasion (LVSI), involvement of the cervix, adnexa, parametrium, and lymph nodes (LNs) were noted. Follow-up periods, presence of recurrence, disease-specific death, overall mortality, and overall survival rates were recorded. All preoperative serum CA 125 levels were measured by our institutional laboratory using a chemiluminescence microparticle immunoassay technique on an Architect i2000 SR Immunoassay Analyzer (Abbott, Abbott Park, IL, USA). To determine the association between the preoperative serum CA 125 level and prognostic factors, the cut-off value for the CA 125 level was accepted as 16 IU/L, based on the recent literature [17,22–24].

### 2.1. Statistical analysis

The data were expressed as the mean ± standard deviation or median and range for continuous variables, and binary variables were reported as the number and percentage. Comparison of the continuous variables between the groups was performed using the nonparametric Mann-Whitney U test for all of the different clinicopathologic factors, except the grade. The Kruskal-Wallis test was used for comparison of the CA 125 levels in terms of the grade. The chi square or Fisher exact tests, where applicable, were used to determine the association of CA 125 levels with the clinicopathologic variables (categorical variables). The Kolmogorov-Smirnov test was used to test for normality of the distribution.

To determine the sensitivity and specificity of the various cut-off levels, receiver operating characteristic (ROC) curve analysis was performed for associated factors confirmed by logistic regression analysis. Time of follow-up was measured from the date of diagnosis to the date of the last visit or death. Disease-specific survival was calculated as the number of years from the first treatment to the date of death as the result of endometrium carcinoma, or the last follow up date for patients who were still alive. Kaplan-Meier survival analysis was used to compare survival outcomes in the CA 125 groups. P < 0.05 was accepted as statistically significant. All analyses were performed using SPSS Statistics for Windows 17.0 (SPSS Inc., Chicago, IL, USA).

## 3. Results

The mean follow-up period was 25.5 (range: 4–93) months. During this period, 7 patients (2.7%) died. The overall mortality rate for the whole group was 4.7% (n = 12). The mean age of the patients was 61 ± 9.3 years, and 31 (12.1%) patients were <50 years of age, while 209 (81.6%) were postmenopausal. The mean body mass index (BMI) was 34.3 ± 7.4 kg/m2 and median tumor size was 3.5 (range: 1–12) cm. The demographic characteristics of the cohort are summarized in Table 1. While 252 patients (98.4%) had endometrioid EC, 4 (1.6%) had mucinous EC. The median and mean CA 125 were 16 (range: 4–1933) IU/L and 47.8 ± 154, respectively. 

**Table 1:** Baseline and disease characteristics of the cohort.

Grade, n (%) Grade 1Grade 2Grade 3	132 (51.6)93 (36.3)31 (12.1)
Stage, n (%)Stage IStage IIStage IIIStage IV	211 (82.4)10 (3.9)30 (11.7)5 (2)
Type of surgery, n (%)	
LaparoscopyLaparotomy	143 (55.9) 113 (44.1)
Hysterectomy Hysterectomy + systematic lymphadenectomy	43 (16.8) 213 (83.2)
Median number of LNs removed, n (range)- Median number pelvic LNs- Median number of paraaortic LNs	55 (1–164) 29 (6–65) 24 (1-94)
Adjuvant therapy, n (%) - Radiotherapy - Chemotherapy- Combined therapy* Sandwich therapy	99 (38.7)58 (41.1)13 (9.2)28 (25.9)*12 (12.1)

The median serum CA 125 levels were significantly higher in patients with >50% MI, LN metastasis, positive LVSI, cervical stromal and adnexal involvement, advanced stage, positive peritoneal cytology, recurrence, and adjuvant therapy requirement. There was no statistical difference in the median serum CA 125 levels for different histologic grades and disease-specific death (Table 2). To define the prognostic role of the serum CA 125, cut-off values were determined using ROC curve statistical tests with clinicopathological factors. These CA 125 cut-off values ranged from 15.3 to 22.9 IU/L (sensitivity 61%–77%, specificity 52%–73%) (Table 3). 

**Table 2:** Median CA 125 levels based on different clinicopathologic factors.

Clinicopathologic factors		N (%)	Median CA 125 level (IU/L) (range)	P-value
Grade	1	132 (51.6)	14.5 (4–600)	0.052
	2	93 (36.3)	16 (4–811.7)	
	3	31 (12.1)	20 (5–1933)	
MI(>50%)	+	56 (22.3)	19.5 (5–788)	0.007
	–	195 (77.7)	15 (4–1933)	
Lymph node metastasis	+	22 (8.6)	31 (7–788)	<0.001
	–	234 (91.4)	15 (4–1933)	
LVSI	+	79 (30.9)	20 (4–1933)	<0.001
	–	177 (69.1)	13 (4–811.7)	
Cervical stromal invasion	+	20 (7.8)	20.8 (4–788)	0.017
	–	236 (92.2)	15 (4–1933)	
Adnexal involvement	+	26 (10.4)	35 (6-1933)	<0.001
	–	225 (89.6)	15 (4–600)	
Positive peritoneal cytology	+	9 (4)	33 (7–1933)	0.029
	–	218 (96)	15 (4–600)	
Advanced stage(stage 3–4)	+	45 (17.6)	24 (4–1933)	<0.001
	–	211 (82.4)	15 (4–600)	
Recurrence	+	100 (39.1)	19 (4–1933)	0.002
	–	156 (60.9)	13.5 (4–600)	
Adjuvant therapy requirement	+	18 (7)	21.3 (7–1933)	0.049
	–	238 (93)	15 (4–788)	
Disease-specific death	+	7 (2.7)	19.5 (13–444)	0.050
	–	249 (97.3)	15 (4–1933)	

**Table 3:** Preoperative CA 125 cut-off values, sensitivity, specificity, AUC (95% CI) in type 1 endometrium cancer at different clinicopathologic factors.

	Cut-off value	Sensitivity, specificity	AUC (95%CI)	P-value
Advanced grade (grade 3)	15.8 IU/L	61%–52%	0.60 (0.49–0.71)	0.07
MI (>50%)	16.1 IU/L	61%–57%	0.62 (0.53–0.71)	0.007
Lymph node metastasis	22.9 IU/L	76%–72%	0.76 (0.65–0.87)	<0.001
LVSI	16.7 IU/L	62%–61%	0.68 (0.60–0.75)	<0.001
Cervical stromal invasion	18.7 IU/L	70%–63%	0.66 (0.53–0.80)	0.017
Adnexal involvement	18.7 IU/L	77%–64%	0.77 (0.67–0.87)	<0.001
Positive peritoneal cytology	22.9 IU/L	67%–73%	0.72 (0.50–0.93)	0.029
Advanced stage (stage 3-4)	18.7 IU/L	69%–66%	0.71 (0.61–0. 80)	<0.001
Adjuvant therapy requirement	15.3 IU/L	62%–57%	0.62 (0.54–0.69)	0.002
Recurrence	18.4 IU/L	61%–61%	0.64 (0.49–0.78)	0.049
Disease-specific death	18.4 IU/L	71%–61%	0.72 (0.57–0.87)	0.05

In agreement with this CA 125 cut-off value, the statistical analysis showed a significant relation between the CA 125 value and MI, advanced stage, LN metastasis, LVSI, cervical stromal and adnexal involvement, adjuvant therapy requirement with P = 0.023, P < 0.001, P = 0.003, P = 0.001, P = 0.009, P = 0.001, and P = 0.003, respectively (Table 4). On the other hand, the statistical analysis failed to show a significant relation between the CA 125 value and tumor grade, positive peritoneal cytology, recurrence, and disease-specific death rate (Table 4). Patients with a CA 125 value ≥16 IU/L had a significantly lower 5-year disease-specific survival rate when compared to patients with a CA 125 value <16 IU/L (95.4% vs. 98.9%, P = 0.047) (Figure).

**Table 4:** Comparison of characteristics of patients with type 1 endometrial cancer with and without elevated CA 125 when cut-off is16 IU/L.

	CA 125 level < 16 IU/L, n (%)	CA 125 level ≥ 16 IU/L, n (%)	P-value
Advanced grade (grade 3)	12/124 (9.7)	19/126 (15.1)	0.25
MI (>50%)	20/124 (16.1)	36/127 (28.3)	0.023
Lymph node metastasis	3/99 (3)	18/121 (14.9)	0.003
LVSI	26/127 (20.5)	53/129 (41.1)	0.001
Cervical stromal invasion	4/127 (3.1)	16/129 (12.4)	0.009
Adnexal involvement	5/124 (4)	21/127 (14.8)	0.001
Positive peritoneal cytology	2/115 (1.7)	7/112 (6.3)	0.09
Advanced stage (stage 3–4)	11/127 (8.7)	34/129 (25.3)	<0.001
Adjuvant therapy requirement	38/127 (29.9)	62/129 (47.3)	0.003
Recurrence	6/127 (4.7)	12/129 (9.3)	0.22
Disease-specific death	1/127 (0.8)	6/129 (4.7)	0.12

**Figure:**
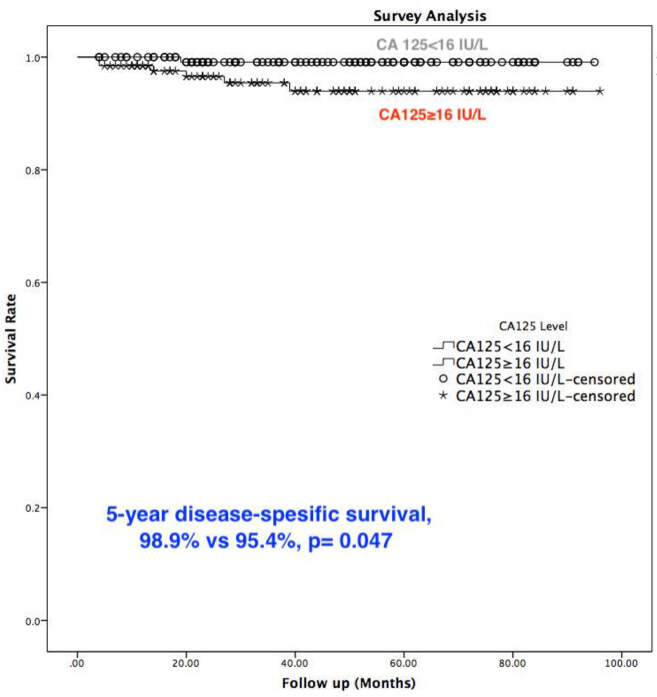
Disease-specific survival of the type 1 endometrial cancer patients with and without elevated CA 125 when cut-off is16 IU/L.

## 4. Discussion

Type 1 endometrial cancer is the most common tumor in gynecology, which mostly presents in the early stages and has a relatively lower rate of recurrence when compared to type 2 endometrial cancer or uterine sarcomas. In surgically-treated patients, an adjuvant strategy is based on the histological type and grade, as well as tumor stage [3]. In the current study, it was aimed to evaluate the prognostic role of CA 125, and define cut-off values for clinicopathologic factors. ROC-curve analysis was used for each historical prognostic hallmark to define the proper cut-off value for the whole group. Based on the literature, a cut-off value of 16 IU/L was defined [17,22–24]. When this cut-off value was used, a significant relation was found between the CA 125 value and MI, advanced stage, LN metastasis, LVSI, cervical stromal, and adnexal involvement. Furthermore, it is noteworthy that the CA 125 value showed a significant effect on the 5-year disease-free survival time. This statistically significant difference reached an absolute difference of 3.5% (95.4 vs. 98.9%) in the Kaplan-Meier survival analysis, which has high clinical relevance.

In the literature, a respective number of studies have mostly included whole histological types of endometrial cancer and reported that elevated serum CA 125 levels might be useful in determining poor prognostic factors, such as extrauterine spread and LN metastasis in EC [25–27]. There have been different CA 125 cut-off values reported in different studies [8,14,16,17]. A study that involved 757 patients, which defined age-stratified cut-off levels for CA 125 values to improve prognostic classification in EC, suggested that the cut-off value was 35 IU/L for patients >49 years of age, and 105 IU/L for those <49 [8]. On the other hand, cut-off levels of poor prognostic factors, which were defined using the ROC curve, ranged from 16.2 to 40.8 IU/L, with 53.4%–84.2% sensitivity and 43.9%–81.7% specificity, in the study of Kim et al. [17]. Previous studies have also reported preoperative serum CA 125 cut-off values in the range of 15 to 35 IU/L for poor prognostic factors in EC [11,14,15]. Based on the current results, the serum CA 125 cut-off values, which were determined according to the clinicopathologic factors, ranged from 15.3 to 22.9 IU/L (sensitivity 61%–77%, specificity 52%–73%). This study specifically consisted of patients with type 1 EC, which may have been a possible reason for the lower cut-off values when compared to those in the study of Chao et al., which included all types of EC [8]. Moreover, 12.1% of the patients were <50 years of age in the cohort herein, therefore, no further analysis was performed for this age group. In a study that included 423 patients with endometrioid EC, elevated CA 125 levels (35 IU/L) were found to be associated with tumor spreading and LN metastasis; however, after adjusting for age, the CA 125 cut-off to predict LN metastasis was found to be 16 IU/L in patients >50 years of age, which was similar to the results herein [24].

Moreover, the current study demonstrated that the median serum CA 125 levels were significantly higher in patients with adverse clinicopathologic factors, except for advanced grade. The studies of Kim et al. and Chung et al. did not find a relationship between higher CA 125 levels and grade, similar to the current results [14,17]. There was no statistical difference in the median serum CA 125 levels for disease-specific death. However, the median follow-up in the current study was 25.5 months, while the mortality rate related to the disease was 7/256 (2.7%), and these results may differ as the follow-up period increases. The results of the present study demonstrated that deep MI, LN metastasis, LVSI, cervical stromal invasion, adnexal involvement, advanced stage, and adjuvant treatment requirements were higher in patients with a CA 125 level ≥16 IU/L than in those with a CA 125 level <16 IU/L, which indicated a lower cut-off value (16 IU/L), instead of the conventional 35 IU/L cut-off in preoperative CA 125 testing. Moreover, defining the preoperative CA 125 cut-off value as 16 IU/L provided higher rates of disease-specific survival in the group with a CA level 125 <16 IU/L. However, it was shown that the preoperative serum CA 125 levels themselves were not sufficient to predict positive peritoneal cytology, recurrence, and disease-specific death; therefore, additional diagnostic methods, such as imaging studies, are required for preoperative counseling of patients with EC. 

Routine intraoperative frozen evaluation has been performed to decide systematic lymphadenectomy in patients with a grade 1–2 tumor, tumor size <2 cm, and MI <50% in recent years. Moreover, no strict criteria have been assigned in grade 2 EC cases. Actually, the definitions of patients at low- and high-risk were derived from the findings of final pathologic examinations. DiSaia and Creasman supported the staging of all grade 2 lesions, whether invasion was present or not [28]. On the contrary, Cochrane found no evidence that lymphadenectomy decreased the risk of death or disease recurrence when compared with not having a lymphadenectomy in women with presumed stage I disease [29]. However, it is known that 15% of histologic grade 2 tumors are reported as grade 3 tumors in final pathology [30]. Hence, there is still a discrepancy between the clinical guidelines and the actual practice adopted by clinicians regarding the role of lymphadenectomy in EC. In this context, based on the experience of the surgeon, lymphadenectomy was performed on patients with grade 2 tumors to avoid undertreatment in our department. 

The current study had some limitations, such as its retrospective design and selection bias. Moreover, patients without preoperative CA 125 levels were not included. However, the fact that only type 1 EC was included in this study allowed it to be specific. Additionally, the standard procedures performed by the same team and maintaining the follow-ups of patients with a multidisciplinary approach were the advantages of the study. 

## 5. Conclusion

The data showed that choosing a lower threshold value for the CA 125 level (16 IU/L), instead of 35 IU/L, could be more useful in type 1 EC patients with negative prognostic factors. Therefore, preoperative serum CA 125 testing should be integrated into routine preoperative evaluation of EC. However, further studies are needed to identify the optimal cut-off level of CA 125.

## Conflict of interest

The authors declare that there are no conflicts of interest.
